# Causal associations between gut microbiota and autism spectrum disorder and attention-deficit/hyperactivity disorder: a two-sample mendelian randomization study

**DOI:** 10.3389/fgene.2026.1972696

**Published:** 2026-10-16

**Authors:** Shangyun Shi, Ancha Baranova, Hongbao Cao, Fuquan Zhang

**Affiliations:** 1 Department of Psychiatry, The Affiliated Brain Hospital of Nanjing Medical University, Nanjing, China; 2 School of Systems Biology, George Mason University, Fairfax, VA, United States; 3 Vavilov Institute of General Genetics, Russian Academy of Sciences, Moscow, Russia; 4 Institute of Neuropsychiatry, The Affiliated Brain Hospital of Nanjing Medical University, Nanjing, China

**Keywords:** attention deficit hyperactivity disorder, autism spectrum disorder, brain-gut axis, gut microbiota, mendelian randomization

## Abstract

**Background:**

The gut microbiota have been implicated in the development of autism spectrum disorder (ASD) and attention-deficit/hyperactivity disorder (ADHD). However, the precise causal relationships between the gut microbiota and ASD or ADHD remain unclear.

**Methods:**

We investigated potential causal associations between gut microbiota and two neurodevelopmental mental disorders (ASD and ADHD) via Mendelian randomization (MR) analyses. We used genome-wide association study (GWAS) summary datasets, including data from the MiBioGen consortium (N = 18,340), the Dutch Microbiome Project (N = 8,208), an ASD GWAS (N = 46,350), and an ADHD GWAS (N = 292,548).

**Results:**

Our MR analysis identified 16 gut bacterial taxa associated with ASD. Seven taxa were associated with an increased risk (OR 1.03–1.10), while nine were associated with a decreased risk (OR 0.89–0.96). Conversely, 28 gut bacterial taxa were causally linked to ADHD: 16 were associated with an increased risk (OR 1.02–1.04), and 12 were associated with a decreased risk (OR 0.95–0.98). Both ASD and ADHD were causally influenced by the family “Clostridiales noname,” but with opposite effects.

**Conclusion:**

Our MR analysis revealed causal effects of specific gut microbiota on ASD and ADHD, suggesting that certain gut microbes may play a role in the development and treatment of these disorders, as well as in their differential diagnosis.

## 1 Introduction

Two common neurodevelopmental disorders of childhood, autism spectrum disorder (ASD) and attention-deficit/hyperactivity disorder (ADHD), are steadily increasing in prevalence (Lyall et al., 2017; Thapar and Cooper, 2016). ASD is characterized by social communication deficits and the presence of restricted, repetitive behaviors or interests (Hirota and King, 2023). The core features of ADHD are developmental delays, inattention, hyperactivity, and impulsivity, and these difficulties often persist into adulthood (Thapar and Cooper, 2016).

People with ASD and ADHD require a significant amount of care and financial resources, placing a heavy economic burden on society and their families (Gordon-Lipkin et al., 2018). Both disorders are highly heritable (Antshel and Russo, 2019), and their etiology is influenced by genetic and environmental risk factors. Prenatal and postnatal factors (e.g., low birth weight, preterm birth, certain maternal medications) also contribute, and exposures to environmental toxins are common (Posner et al., 2020; Sciberras et al., 2017; Sharma et al., 2018). Notably, gastrointestinal symptoms are common in ASD and ADHD: up to 70% of individuals with ASD experience constipation, diarrhea, and abdominal pain, and symptom severity correlates with ASD severity. Individuals with ADHD also report gastrointestinal problems (Buie et al., 2010; Vargason et al., 2019; Kedem et al., 2020).

In recent years, researchers have discovered that the gut microbiota regulates the central nervous system through the brain-gut axis and is involved in the occurrence and development of many neurological diseases (Morais et al., 2021; Cryan et al., 2019; Cryan and Dinan, 2012; Koppula et al., 2025; Yang et al., 2025). The composition of the gut microbiota varies widely from birth to adulthood. The initial shaping of the fetal microbiome may take place through placental microbial transfer in the course of intrauterine life (Collado et al., 2016). After natural delivery or cesarean section, the gut is further colonized through vaginal or postnatal microbial contact (Jiménez et al., 2008; Principi and Esposito, 2016). The composition of the microbiome is further influenced by exposure to antibiotics, infections, feeding mode, stress, diet, and host genetics (Nicholson et al., 2012; Tamburini et al., 2016; Kushak et al., 2016). In patients with ASD and ADHD, the composition of the gut microbiome is significantly different from that of non-diseased individuals (Pang et al., 2023; Bundgaard-Nielsen et al., 2023; Prehn-Kristensen et al., 2018). Moreover, longitudinal prospective studies on early-life microbiomes show that microbiome and metabolite signatures are associated with the risks of both ASD and ADHD (Ahrens et al., 2024).

Sharon et al. found that mice receiving gut microbes collected from humans with ASD began to display ASD-like behaviors (Sharon et al., 2019). Talge et al. reported that the increased risk of ADHD observed in children delivered by cesarean section is associated with reduced gut microbiota diversity (Talge et al., 2016). The incidence of ADHD is lower in breastfed children than in non-breastfed children (Pacheco et al., 2015). Dietary interventions and fecal microbiota transplantation have been reported to improve symptoms of ASD and ADHD (Kang et al., 2017; Kwak et al., 2023). Collectively, these findings suggest a potential connection between gut microbiota composition and the presence of ASD or ADHD.

ASD and ADHD are highly comorbid; approximately 20%–50% of children with ADHD meet diagnostic criteria for ASD, and 30%–80% of individuals with ASD meet criteria for ADHD (Rommelse et al., 2010). This frequent comorbidity complicates diagnosis and treatment. To date, associations between the gut microbiota and ASD or ADHD are primarily supported by observational studies (Bundgaard-Nielsen et al., 2023). Causal effects of gut microbes on neurodevelopmental processes remain speculative (Wang et al., 2023). To explore the causality between microbial composition phenotypes and the two mental disorders, ASD and ADHD, Mendelian randomization (MR) is a method of choice.

MR studies employ genetic variants as instrumental variables to evaluate the causal effects of risk factors on specific outcomes. Similar to random assignment in clinical trials, the random assortment of genetic variants in an individual’s diploid genome provides a framework for assessing the causality of observed associations. In MR, associations between exposures and outcomes are inferred from aggregated data in genome-wide association studies (GWAS). This approach minimizes the effects of confounding factors and reverse causation (Lawlor et al., 2008a; Lawlor et al., 2008b) and is now widely used to infer causality from a genetic perspective (Fu et al., 2025; Zhao et al., 2024a; Zhou et al., 2024; Zhao et al., 2024b; Jiang et al., 2025; Luo et al., 2024). In this study, we performed a two-sample MR (TSMR) analysis to explore the causal effects of various gut microbiota constituents on two mental disorders, ASD and ADHD. The analysis revealed causal effects of specific gut microbiota on both disorders, suggesting that certain microbial taxa may play a role in their development, treatment, and differential diagnosis.

## 2 Methods

### 2.1 GWAS summary datasets

The GWAS summary results used for this analysis were all derived from publicly available data. The data for ASD, sourced from Grove et al., included 18,381 cases and 27,969 controls (Grove et al., 2019). ADHD dataset with 38,691 cases and 275,986 controls was acquired from Demontis et al. (Demontis et al., 2023). The summary data on the gut microbiota were obtained from two sources: the international consortium MiBioGen (MiBioGen) and the Dutch Microbiome Project (Dutch). MiBioGen (Kurilshikov et al., 2021) comprises GWAS summary statistics from 18,340 participants over 11 countries, covering a total of 211 taxa across 35 families, 20 orders, 16 classes, 9 phyla, and 131 genera. The Dutch (Lopera-Maya et al., 2022) subset data mainly stems from the Dutch Microbiome Project, which investigated the gut microbiome composition and function in 8,208 individuals. From the latter dataset, we utilized GWAS data for 207 taxa (5 phyla, 10 classes, 13 orders, 26 families, 48 genera, and 105 species). These two microbial datasets do overlap by about 30%. All participants were of European descent. Ethical approval was obtained in all original studies.

### 2.2 Instrumental variable selection

Single-nucleotide polymorphisms (SNPs) were selected as instrumental variables (IVs) at genome-wide significance thresholds of p < 5 × 10^−8^ and p < 1 × 10^−5^. To ensure independence, SNPs were pruned using r^2^ < 0.001 within a 10 Mb window based on the 1000 Genomes Project (European reference panel). Effect alleles were harmonized across exposure and outcome datasets. The variance explained by the selected instrumental SNPs was quantified using the R^2^ statistic. All retained SNPs yielded an F-statistic >10, precluding weak instrument bias (Burgess and Thompson, 2011).

### 2.3 MR analysis

In this study, we used three different models from the TwoSampleMR package (https://mrcieu.github.io/TwoSampleMR) in R software (version 4.0.5) to investigate causal relationships. We primarily used the inverse-variance-weighted (IVW) model, which assumes a zero intercept and estimates causal effects via fixed-effect meta-analysis. To ensure the robustness of our findings, we conducted sensitivity analyses using additional MR methods, such as the weighted median (WM) and MR-Egger, which differ from IVW in their assumptions. The latter model, for instance, assumes that pleiotropic effects are independent and employs weighted linear regression of outcome coefficients against exposure coefficients. We assessed the presence of pleiotropy by examining the intercepts of MR-Egger regression, which provided insights into mean-level pleiotropy. Furthermore, we evaluated the heterogeneity of our results using both I^2^ statistics and Cochran’s Q test, considering values where both I^2^ > 0.25 and P < 0.05 as indicative of significant heterogeneity. An IVW-based P < 0.05 indicated a significant correlation between the gut microbiota and the two mental disorders. In addition, we applied the Benjamini–Hochberg false discovery rate (FDR) correction to the P-values for all tested associations between gut microbiota and the two disorders. The flowchart of the current study is depicted in Figure 1.

**FIGURE 1:**
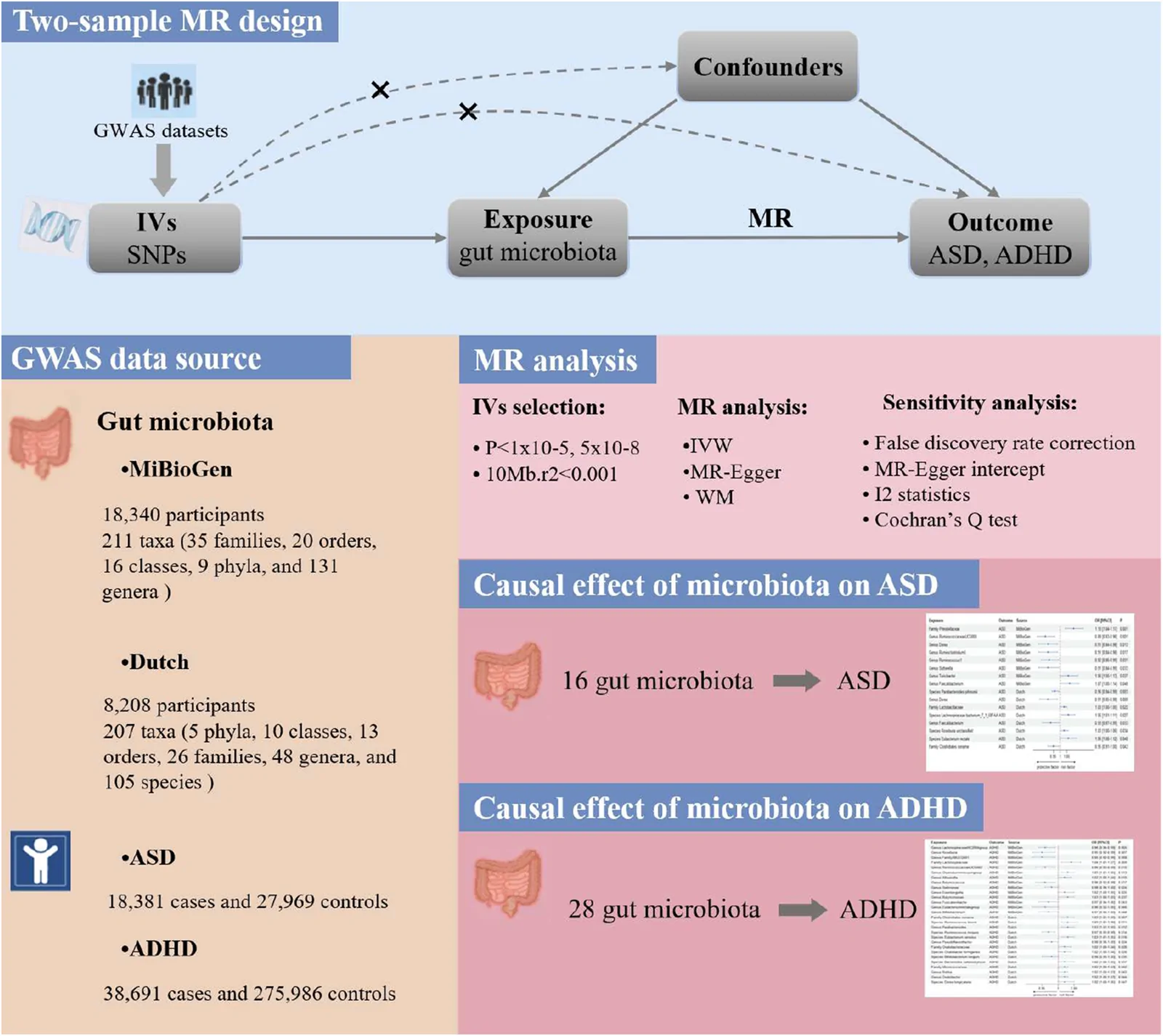
Study flowchart. GWAS: genome-wide association study; IV: instrumental variable; SNP: single-nucleotide polymorphism; MR: Mendelian randomisation; ASD: autism spectrum disorder; ADHD: attention deficit hyperactivity disorder; MiBioGen: MiBioGen alliance; Dutch: Dutch Microbiome Project; IVW: inverse variance weighted; WM: weighted median.

## 3 Results

Using IVW, a total of 16 bacterial clades were identified as conferring causal effects on ASD. Among these, family Prevotellaceae was significantly and positively associated with the risk of ASD (OR: 1.03–1.10, P ≤ 0.046), while genus RuminococcaceaeUCG005 and genus Dorea (OR: 0.89–0.96, P ≤ 0.043) were highlighted as protective factors for this condition (Table 1; Figure 2).

**TABLE 1:** Causal effects of the gut microbiota on ASD.

Exposure	Outcome	Source	Or [95%CI]	P
Family prevotellaceae	ASD	MiBioGen	1.10 [1.04–1.17]	1.12E-03
Genus RuminococcaceaeUCG005	ASD	MiBioGen	0.89 [0.83–0.96]	1.25E-03
Genus dorea	ASD	MiBioGen	0.91 [0.84–0.98]	0.013
Genus Ruminiclostridium5	ASD	MiBioGen	0.91 [0.84–0.98]	0.017
Genus Ruminococcus1	ASD	MiBioGen	0.92 [0.86–0.99]	0.031
Genus sutterella	ASD	MiBioGen	0.91 [0.84–0.99]	0.033
Genus turicibacter	ASD	MiBioGen	1.06 [1.00–1.13]	0.037
Genus faecalibacterium	ASD	MiBioGen	1.07 [1.00–1.14]	0.046
Species parabacteroides johnsonii	ASD	Dutch	0.96 [0.94–0.99]	2.61E-03
Genus dorea	ASD	Dutch	0.91 [0.85–0.98]	8.83E-03
Family lactobacillaceae	ASD	Dutch	1.03 [1.00–1.05]	0.022
Species lachnospiraceae bacterium_7_1_58FAA	ASD	Dutch	1.06 [1.01–1.11]	0.027
Genus faecalibacterium	ASD	Dutch	0.93 [0.87–0.99]	0.033
Species roseburia unclassified	ASD	Dutch	1.03 [1.00–1.06]	0.034
Species eubacterium rectale	ASD	Dutch	1.06 [1.00–1.12]	0.04
Family clostridiales noname	ASD	Dutch	0.95 [0.91–1.00]	0.043

ASD: autism spectrum disorder; CI: confidence interval; OR: odds ratio.

**FIGURE 2:**
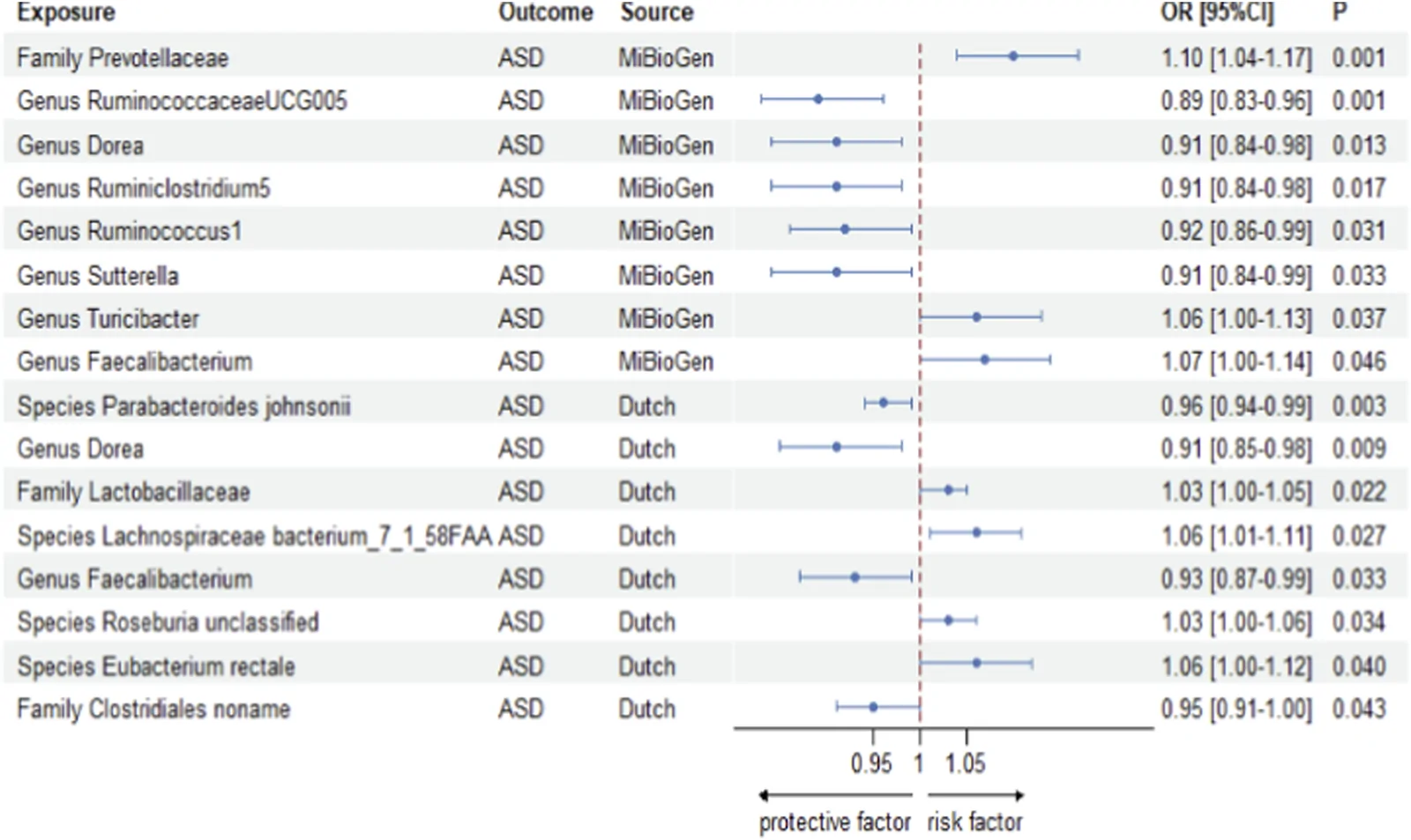
Causal effects of the gut microbiota on ASD. ASD: autism spectrum disorder; CI: confidence interval; OR: odds ratio; MiBioGen: gut microbiota data from the MiBioGen international consortium; Dutch: gut microbiota data from the Dutch Microbiome Project.

A total of 28 taxa of gut microbiota were associated with ADHD. Including genus LachnospiraceaeNC2004group and genus Roseburia, etc., conferring protection against ADHD (OR: 0.95–0.98, P ≤ 0.048), and family Lachnospiraceae (OR: 1.02–1.04, P ≤ 0.047) promoting this condition (Table 2; Figure 3).

**TABLE 2:** Causal effects of the gut microbiota on ADHD.

Exposure	Outcome	Source	Or [95%CI]	P
Genus LachnospiraceaeNC2004group	ADHD	MiBioGen	0.96 [0.94–0.99]	5.11E-03
Genus roseburia	ADHD	MiBioGen	0.95 [0.92–0.99]	6.80E-03
Genus FamilyXIIIUCG001	ADHD	MiBioGen	0.95 [0.92–0.99]	8.17E-03
Family lachnospiraceae	ADHD	MiBioGen	1.04 [1.01–1.07]	9.10E-03
Genus RuminococcaceaeUCG003	ADHD	MiBioGen	0.96 [0.93–0.99]	0.01
Genus clostridiuminnocuumgroup	ADHD	MiBioGen	1.03 [1.01–1.05]	0.013
Genus allisonella	ADHD	MiBioGen	1.02 [1.00–1.04]	0.015
Genus butyricicoccus	ADHD	MiBioGen	0.96 [0.93–0.99]	0.017
Genus sellimonas	ADHD	MiBioGen	0.98 [0.96–1.00]	0.024
Genus eisenbergiella	ADHD	MiBioGen	1.02 [1.00–1.05]	0.025
Genus butyricimonas	ADHD	MiBioGen	1.03 [1.00–1.05]	0.037
Genus fusicatenibacter	ADHD	MiBioGen	0.97 [0.94–1.00]	0.043
Genus eubacteriumrectalegroup	ADHD	MiBioGen	0.96 [0.92–1.00]	0.046
Genus bifidobacterium	ADHD	MiBioGen	0.97 [0.94–1.00]	0.048
Family clostridiales noname	ADHD	Dutch	1.03 [1.01–1.05]	6.96E-03
Species ruminococcus bromii	ADHD	Dutch	1.03 [1.01–1.06]	0.011
Genus parabacteroides	ADHD	Dutch	1.03 [1.01–1.05]	0.012
Species ruminococcus torques	ADHD	Dutch	0.97 [0.95–0.99]	0.014
Species eubacterium ramulus	ADHD	Dutch	1.03 [1.01–1.05]	0.016
Genus pseudoflavonifractor	ADHD	Dutch	0.98 [0.96–1.00]	0.024
Family oxalobacteraceae	ADHD	Dutch	1.02 [1.00–1.04]	0.026
Species oxalobacter formigenes	ADHD	Dutch	1.02 [1.00–1.04]	0.026
Species bifidobacterium longum	ADHD	Dutch	0.98 [0.95–1.00]	0.035
Species *Bacteroides* cellulosilyticus	ADHD	Dutch	1.02 [1.00–1.05]	0.037
Family micrococcaceae	ADHD	Dutch	1.02 [1.00–1.03]	0.042
Genus rothia	ADHD	Dutch	1.02 [1.00–1.03]	0.042
Genus oxalobacter	ADHD	Dutch	1.02 [1.00–1.03]	0.044
Species dorea longicatena	ADHD	Dutch	1.02 [1.00–1.05]	0.047

ADHD: attention deficit hyperactivity disorder; CI: confidence interval; OR: odds ratio.

**FIGURE 3:**
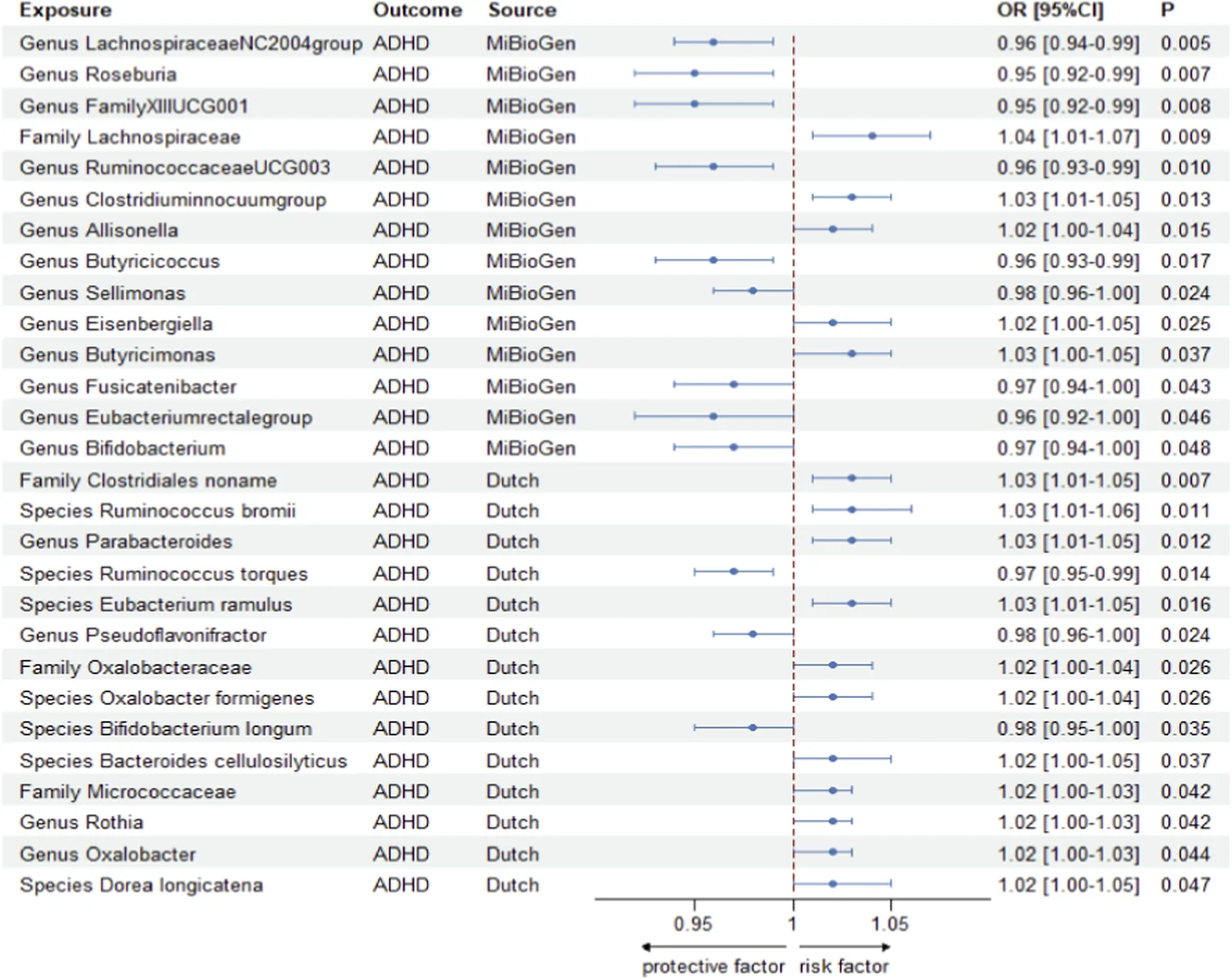
Causal effects of the gut microbiota on ADHD. ADHD: attention deficit hyperactivity disorder; CI: confidence interval; OR: odds ratio; MiBioGen: gut microbiota data from the MiBioGen international consortium; Dutch: gut microbiota data from the Dutch Microbiome Project.

Notably, genetic factors associated with an increased abundance of the unnamed Clostridiales family were linked to a reduced risk of ASD and an increased risk of ADHD.

After FDR correction, none of the associations retained an FDR below 0.05. Cochran’s Q-test indicated no significant heterogeneity among the SNPs (Q_P > 0.05). However, MR-Egger analysis indicated evidence of horizontal pleiotropy for the genus Faecalibacterium (p_pleiotropy <0.05). Therefore, this result should be interpreted with caution (Supplementary Tables S1-S4).

## 4 Discussion

Previous studies suggested that the intestinal microbiota may influence ASD and ADHD through the well-known but still elusive gut-brain axis (Ahrens et al., 2024; Gonçalves et al., 2024). Although the relationships identified between gut microbial species and neurodevelopmental phenotypes in observational studies are difficult to assess for causality, MR analysis may provide a clearer view of the complex interactions between the gut and the brain.

The gut microbiota may participate in the functioning of the brain-gut axis through endocrine, neuronal, and immune signaling pathways and thereby directly influence the symptoms of neurological diseases (Margolis et al., 2021). Numerous studies have implicated microbial production of neurotransmitters and metabolites, including dopamine, serotonin, gamma-aminobutyric acid, and short-chain fatty acids (SCFAs) in both ASD and ADHD (Wang et al., 2023; Boonchooduang et al., 2020; Koh et al., 2016; Fattorusso et al., 2019).

Here, MR analysis pointed to a causal relationship between genetic predisposition and a higher abundance of the genus LachnospiraceaeNC2004group and genus Roseburia, etc., as a causal protective factor against ADHD (OR: 0.95–0.98, P ≤ 0.048), while at the family level, Lachnospiraceae (OR: 1.02–1.04, P ≤ 0.047) causally promoted this condition. In one study, Lachnospiraceae bacteria were found to be significantly reduced in the ADHD group at the species level (Wan et al., 2020), while other Lachnospiraceae, including UCG-010, were overrepresented in the gut of patients with ADHD (Wang et al., 2022). Notably, in earlier studies, the products of bacterial metabolism by some of the Lachnospiraceae have been associated with the etiology of depression (Cheung et al., 2019). Specifically in ADHD, a significant association was noted between withdrawal and depression symptoms and the abundance of one of the Lachnospiraceae species after adjusting for sex, age, and the ADHD core symptoms score (Lee et al., 2022). Interestingly, genetic classification places major depressive disorder (MDD) in the same group of psychiatric disorders as ADHD and ASD (Cao et al., 2022; Wu et al., 2020), raising the question of whether this genetic grouping is reflected, at least in part, by overlapping features of their microbiomes.

Our results show that the genetic factors contributing to an increase in the abundance of the family Prevotellaceae significantly contribute to an increase in the risk of ASD. These strong and causal ties that we have detected between the family Prevotellaceae and ASD are consistent with observations made in previous studies (Dan et al., 2020; Li et al., 2023). Prevotellaceae produce propionate (Ramos Meyers et al., 2022; Louis and Flint, 2017). This impairs social behavior as well as induces cognitive impairment, sensorimotor dysfunction, ASD-like symptoms, and promotes reactive astrocytosis in rats (Shultz et al., 2015; Shultz et al., 2008). Moreover, the observed effects are dose-dependent (Meeking et al., 2020). An increase in the number of reactive astrocytes may lead to an imbalance of neurotransmitters, as well as impede nerve signaling, alter blood-brain barrier permeability, and affect the function of the nerve cells (Pekny and Nilsson, 2005; Kumar et al., 2023; Nam et al., 2023; Yousefi et al., 2022).

When the majority of propionate is metabolized into butyrate, these adverse effects may be reversed or alleviated. Both intestinal butyrate infusions and *in vitro* application of butyrate to cultured myenteric ganglia positively affect the enteric nervous system (ENS) by increasing the proportion of cholinergic nerve cells, translating into increased intestinal peristalsis, which is thought to be beneficial to the gut (Koh et al., 2016). In an MR study, an increase in cholinergic signal was found to reduce the risk of ASD (Liu et al., 2022). Conversely, in children diagnosed with ASD, butyrate concentrations are reduced (Liu et al., 2019).

In our analysis, a genetically supported higher abundance of genera RuminococcaceaeUCG005, Dorea, and Ruminiclostridium5 was found to have strong protective effects against ASD, an effect that may be explained by propionate-to-butyrate conversion (Louis and Flint, 2017). Furthermore, the genus Dorea is known to protect intestinal permeability by preventing harmful substances and metabolites from entering the circulatory system (Fu et al., 2017; Zhou et al., 2021). However, our study shows that the genetic signature promoting the growth of a particular species of the genus Dorea, D. longicatena, displays a slightly negative effect on ADHD. For ASD, MR analysis of both GWAS databases confirmed that the genetic components promoting the enrichment of genus Dorea reduce the risk of ASD, consistent with the result of Li et al. (Li et al., 2023). However, for the genus Faecalibacterium, analysis of MiBioGen and the Dutch cohort yielded divergent findings, possibly due to differences in their study populations or the profiling techniques employed. Hence, the role of the genus Faecalibacterium in ASD remains unclear and deserves further study.

Interestingly, the effects of the family Clostridiales noname on ASD and ADHD were opposite of each other. Genetic predisposition to a higher abundance of the family Clostridiales reduces the risk of ASD but significantly increases the risk of ADHD. Butyrate-producing Clostridiales belong to the phylum Firmicutes, class Clostridia. Notably, these bacteria are known to inhibit dopamine-β-hydroxylase, which blocks the conversion of dopamine to norepinephrine. In turn, this may cause a significant increase in dopamine levels while indirectly lowering the levels of norepinephrine (Hamamah et al., 2022), which seems to be associated with attention-seeking and impulsive behavior (Del Campo et al., 2011), which is a hallmark of ADHD. Similarly, SCFA-producing Ruminococcus may exert a protective effect on dopaminergic neurons, which could be counterbalanced by their inflammation–promoting role in mucin degradation. Our MR analysis suggests ADHD-promoting but ASD-protecting roles of the genetic components contributing to the higher abundance of Ruminococcus species. Although their exact effects on dopamine transporters, dopaminergic neurons, and relevant receptors are far from clear, clinical evidence highlights the dysregulation of dopamine metabolism in patients with ASD and ADHD (Leggio et al., 2019). Thus, the effects of dopamine-beta-hydrolase-inhibiting bacteria in these conditions look plausible.

To sum the findings up, the roles of specific microbial strains in the pathogenesis of ASD and ADHD may be disease-specific and causal. These strains may have different effects on the regulation of the brain-gut axis, activation of the immune system, and regulation of neurotransmitters, thereby affecting the development and clinical manifestations of these two disorders. In children’s mental health research, the majority of studies investigating gut microbiota have focused on patients with ASD, while evidence regarding the role of the gut-brain axis in ADHD remains limited. We utilized data from two large-scale gut microbiota GWAS to identify potential causal relationships, which may provide candidate microbial communities for subsequent research, particularly in the context of ADHD. In addition, we also found that there is less overlap in the microbiota that is causally related to these two diseases, which may be helpful for subsequent differential diagnosis research. MR analysis was employed to mitigate confounding factors and reverse causality. Multiple sensitivity analyses were conducted to ensure the robustness of the results.

However, the study has several limitations. All participants were drawn from European populations; thus, caution should be exercised when generalizing the conclusions to other populations. MR only considers the genetic factors influencing the composition of gut microbiota and the two mental disorders; therefore, interpretation of the results should be done cautiously. Gut microbiota may be influenced by other factors such as dietary habits, health status, age, and gender, many of which have a hereditary component, and it is currently uncertain whether genetic tools are associated with these confounding factors or the abundance of bacterial taxa themselves. Moreover, after applying FDR correction across all associations, none remained below 0.05, underscoring the conservative, hypothesis-generating nature of our findings; these associations should therefore be considered suggestive and warrant validation in independent cohorts. Finally, the MiBioGen and Dutch microbiome datasets used in our TSMR analyses partially overlap. Although the bias introduced by sample overlap is expected to be limited given that all instrumental variables had F-statistics greater than 10, its potential influence on the causal estimates cannot be entirely excluded. Independent validation in non-overlapping cohorts is therefore warranted to confirm our findings.

## 5 Conclusion

We identified specific gut microbiota taxa that may be associated with ASD and ADHD, and the two disorders appear to involve distinct microbial communities. These findings provide suggestive evidence for a potential role of the gut microbiota in the development of ASD and ADHD and may inform differential diagnosis and the development of microbiota-targeted interventions.

## Data Availability

The original contributions presented in the study are included in the article/Supplementary Material, further inquiries can be directed to the corresponding author.
